# The Effect of Lipid Metabolism on CD4^+^ T Cells

**DOI:** 10.1155/2021/6634532

**Published:** 2021-01-05

**Authors:** Feiyang Cai, Shuxin Jin, Guangjie Chen

**Affiliations:** Department of Immunology and Microbiology, Shanghai Jiao Tong University School of Medicine, Shanghai Institute of Immunology, No. 280 South Chongqing Rd., Shanghai, China

## Abstract

CD4^+^ T cells play a vital role in the adaptive immune system and are involved in the pathogenesis of many diseases, including cancer, autoimmune diseases, and chronic inflammation. As an important mechanism for energy storage, a lot of researches have clarified that metabolism imbalance interacts with immune disorder, and one leads to the other. Lipid metabolism has close relationship with CD4^+^ T cells. In this review, we discuss fatty acid, cholesterol, prostaglandin, and phospholipid metabolism in CD4^+^ T cell subsets. Fatty acid *β*-oxidation (FAO) is activated in Th17 cell to support the proinflammatory function. Cholesterol promotes Th1, Th2, and Treg cell differentiation. In addition to glucose metabolism, lipid metabolism is also very important for immunity. Here, it is highlighted that lipid metabolism regulates CD4^+^ T cell differentiation and function and is related to diseases.

## 1. Introduction

Different CD4^+^ T cell subsets are involved in various immune responses. Th1 cells are responsible for an increased cell-mediated immune response, especially cellular functions against pathogen infections. Th2 cells mainly activate the humoral immune response against parasites, typically involving immune response in the skin, respiratory tract, and digestive tract. T follicular helper cells (Tfh) cells are located in B cell follicles and are involved in the formation of the germinal center and in B cell selection. Th17 cells are essential for pathogen clearance in the mucosal barrier and are related to the occurrence of autoimmune diseases and inflammatory disorders. Regulatory T (Treg) cells are essential for immune tolerance, and they prevent autoimmune diseases.

In different stages of differentiation, CD4^+^ T cells have varied metabolic characteristics while performing their immune function and are regulated by related signaling pathways. Several recent studies have shown the close relationship between T cell signaling and metabolism. Raptor/mTORC1 signaling in Treg cells promotes cholesterol biosynthesis, which is essential for Treg cell suppressive activity [1]. When cholesterol recycling is impaired, the cholesterol accumulation enhances CD4^+^ T cell activation [2]. Therefore, lipid metabolism plays a vital role in CD4^+^ T cell.

Conventionally, glucose metabolism regulates T cell activation and differentiation [3]. An increasing amount of attention has been drawn to the relationship between lipid metabolism in CD4^+^ T cells and diseases. Cholesterol plays an important role in regulating CD4^+^ T cells, and cholesterol accumulation is relevant to various diseases, such as rheumatoid arthritis (RA) and nonalcoholic steatohepatitis [4, 5]. Hypercholesterolemia mediates CD4^+^ T cell inflammatory response, but it is not completely clear how CD4^+^ T cell together with cholesterol induces long-term inflammatory diseases [6]. Lipoproteins and some derivatives of cholesterol, such as prostaglandins, are also key metabolic regulators of CD4^+^ T cells. They will be precisely summarized in an independent section in this review. Lipid raft, a microdomain that is enriched with cholesterol and sphingophospholipids, assists CD4^+^ T cell activation. Lipid raft affects the geometry and conformation of TCR-MHC cluster and potentially promotes TCR and CD28 engagement [7]. As lipid metabolism regulates T cell response, T cells also influence lipid metabolism. PD-1 ligation in T cells activates fatty acid *β*-oxidation (FAO) by upregulating CPT1A and ATGL [8]. This metabolic mechanism contributes to CD4^+^ T cell differentiation toward Th1 subset. In this review, we focus on how fatty acids, cholesterol, prostaglandins, and phospholipids metabolisms are involved in CD4^+^ T cell function. These insights into lipid metabolism will shed light on the nature how CD4^+^ T cell function is regulated, and how lipid metabolism in CD4^+^ T cells drives immunologic disorders.

## 2. Fatty Acid Metabolism in CD4^+^ T Cells

Fatty acid plays an important role in cell, implicating the synthesis of many essential fatty acid derivatives. Prostaglandin and leukotriene synthesis is dependent on fatty acid as basic material. They are involved in a variety of biology processes, including inflammation and allergy [9]. Effector T cell differentiation depends on *de novo* fatty acid synthesis, and it is heatedly discussed whether FAO is required for Treg cell differentiation [10–12].

### 2.1. *De Novo* Fatty Acid Synthesis in CD4^+^ T Cells

Monounsaturated fatty acids (MUFAs) are required for endogenous fatty acid synthesis, which is catalyzed by stearoyl-CoA desaturases (SCDs). Both human and murine Tfh cells have high SCD expression, and the inhibition of SCDs in a mouse model leads to Tfh cell apoptosis, indicating that fatty acids are critical for Tfh cell survival [13]. MUFA synthesis requires acetyl-CoA and malonyl-CoA. *De novo* fatty acid synthesis takes place in the cytoplasm, so acetyl-CoA is transported out of mitochondria with the mechanism of citrate pyruvate cycle. The key enzyme of *de novo* fatty acid synthesis is acetyl-CoA carboxylase (ACC), which converts acetyl-CoA to malonyl-CoA. Malonyl-CoAs and acetyl-CoAs experience a series of reduction and condensation reaction to form MUFA [14].

Mouse experiments have showed that Th17 cell differentiation depends on ACC1-mediated de novo fatty acid synthesis, which produces phospholipids for the cell membrane [15]. ACC1 deletion suppresses Th17 immune response, proliferation, and infiltration in murine models of colitis [16]. Another study showed that a high-fat diet promotes Th17 cell differentiation and that ACC1 has a similar effect on Th17 cells, including increased IL17A, IL23R, LTB4R1, and CCR6 expression; interestingly, ACC1 modulates ROR*γ*t binding to target genes in Th17 cell differentiation but does not affect ROR*γ*t expression levels [17]. The findings above indicate that there exists a close relationship between Tfh cells, Th17 cells, and *de novo* fatty acid synthesis.

Since a large amount of literature states that glycolysis is required for naïve T cell activation and differentiation, the importance of *de novo* fatty acid synthesis is underestimated [3]. Mamareli et al. have clearly proved that glycolysis alone is not sufficient to support Th17 cell differentiation without the help of *de novo* fatty acid synthesis [16]. Acetyl-CoA that is generated during glycolysis contributes to *de novo* fatty acid synthesis. Thus, glycolysis and *de novo* fatty acid synthesis assists naïve T cell activation and differentiation synergistically, and fatty acid synthesis requires and deserves more attention.

### 2.2. FAO in CD4^+^ T Cell

FAO is the key process in fatty acid degradation and is an important source of ATP. It takes place in the mitochondria, and its key enzyme is carnitine acyl transferase I (CPT1A), which synthesizes acyl carnitine. Fatty acid is activated to be acyl-CoA and CPT1A enables acyl-CoA to enter mitochondria. FAO occurs with a series of reactions, including oxidation, hydration, and decomposition [18].

Dual specificity phosphatase 6- (DUSP6-) depleted Tfh cells produce higher levels of IL-21 due to the activation of mitogen-activated protein kinase (MAPK) signaling, but FAO suppression in these cells resulted in lower levels of IL-21 [19]. This indicates the importance of FAO in MAPK signaling, but the specific lipid that regulates MAPK signaling is unclear. In patients with type 2 diabetes, blocking CPT1A or inhibiting FAO led to less IL-17 production by proinflammatory Th17 cells. This indicates that FAO may activate Th17 inflammation in human type 2 diabetes [20]. Since inflammation contributes to insulin resistance and islet *β* cell dysfunction in type 2 diabetes, suppressing FAO in Th17 cell seems to be an effective way to relieve this disease. In comparison, *de novo* fatty acid synthesis is indispensable for Th17 cell differentiation, but there is little study into whether inhibiting fatty acid synthesis can prevent type 2 diabetes.

It is still controversial whether Treg cell differentiation is dependent on FAO. On the one hand, Th1, Th2, and Th17 cells are more dependent on glycolysis and *de novo* fatty acid synthesis to support effector function, while Treg cells rely more on oxidative phosphorylation and FAO. Fatty acid-binding protein (FABP5) is responsible for fatty acid uptake, and it is highly expressed in Treg cells. *In vitro* studies show that the inhibition of FABP5 in naïve T cells suppresses Treg cell differentiation. The inhibition of FABP5 activates cyclic GMP-AMP synthase (cGAS) stimulator of interferon gene- (STING-) dependent type I IFN signaling, leading to IL-10 production and Treg cell activation [11]. On the other hand, Treg cell differentiation is independent of CPT1A expression, indicating that FAO is not required for Treg cell function [12]. Further studies are needed to clarify the specific lipid molecule or metabolism processing in FAO that is essential for Treg cell differentiation. AMPK activators enhance Treg cell expansion dependent on FAO, while inhibiting Th17 cell differentiation [21]. AMPK activators also enhance mitochondrial generation and fatty acid uptake, suggesting that AMPK signaling helps to provide material and location for FAO, such as fatty acid and mitochondria. Moreover, the impact of AMPK on Treg cell differentiation is mTOR dependent [21]. AMPK directly phosphorylates both tuberous sclerosis complex (TSC2) and raptor to inhibit mTORC1 activity by a dual-pronged mechanism [22]. Therefore, AMPK is likely to inhibit mTORC1 by phosphorylating TSC2 or raptor, thereby promoting Treg cell differentiation.

Thus, FAO plays a vital role in different subsets of CD4^+^ T cells, suggesting that FAO comprehensively regulates T cell metabolism. Moreover, AMPK signaling has the potential to regulate Th17/Treg balance via FAO.

### 2.3. Free Fatty Acid Metabolism in CD4^+^ T Cells

Long-chain fatty acids (LCFAs), including caproic acid, capric acid, and lauric acid, enhance Th1 and Th17 cell differentiation and proliferation via the P38-MAPK pathway, exacerbating the disease in experimental autoimmune encephalomyelitis (EAE) mice, an animal model of multiple sclerosis (MS) [23]. Docosahexaenoic acid (DHA) gets integrated into antigen-activated CD4^+^ T cells and downregulates cytokines related to Th1 and Th17 cells [24]. Researchers studied fat-1 mice, which contain fat-1 to convert n-6 to n-3 polyunsaturated fatty acids (PUFAs). Fat-1 inactivates Th17 cell differentiation by downregulating IL-6 receptor expression. These results showed that n-3 PUFAs suppress Th17 cell differentiation by reducing membrane raft-dependent responsiveness to IL-6 [25]. These studies about LCFAs modify our understanding of inflammation-related diseases, especially MS. The strategy to inhibit LCFAs and the protective role of n-3 PUFAs are potential to optimize the immunomodulatory therapy to fight against the development of MS. In addition, these studies also suggest the prospect of dietary therapy.

Short-chain fatty acids (SCFAs), including acetate, propionate, and butyrate, are produced by gut microbiota-derived bacterial fermentation. Acetate, propionate, and butyrate regulate CD4^+^ T cell differentiation and function via binding of free fatty acid receptor (FFAR), a G-protein-coupled receptor (GPR). Acetate, propionate, and butyrate enhance the phosphorylation of mTOR and STAT3 in Th1 cells, thereby promoting IL-10 production in Th1 cells and relieving colitis [26]. They also activate Treg cell differentiation via FFAR2 and promote IL-10 production to relieve colitis [27]. Acetate, propionate, and butyrate promote Treg cell differentiation by inhibiting the activity of histone deacetylase (HDAC) and enhancing histone H3 acetylation at the promoter of *Foxp3*, thereby promoting the acetylation of *Foxp3* locus, inducing the differentiation of colonic Treg cells and ameliorating the development of colitis. However, researchers did not specify what HDAC is responsible for Treg differentiation [28, 29]. Dietary acetate, propionate, and butyrate expand gut Treg cells by suppressing JNK1 and p38 pathways, thereby lessening the severity of EAE [23]. Compared with Treg cells, Th2 cells upregulate FFAR3 in eosinophilic esophagitis and SCFAs activate Th2 cell differentiation [30]. Thus, extracellular SCFAs also have various effects on CD4^+^ T cell function in inflammatory disease. In summary, the control of LCFAs and utilization of SCFAs lead to a better prognosis in autoimmune diseases.

## 3. Cholesterol in CD4^+^ T Cells

Based on varying densities, lipoproteins are composed of chylomicron, very-low-density lipoprotein, low-density lipoprotein (LDL), and high-density lipoprotein (HDL). LDL and HDL transport endogenous cholesterol from *de novo* cholesterol synthesis and exogenous cholesterol from ingested cholesterol, respectively. CD4^+^ T cells populations also influence cholesterol-related diseases, such as atherosclerosis. Hypercholesterolemia elevates *Foxp3* expression and Treg cell proportion in T cell population. Cholesterol also enhances TCR signaling and aggravates the inflammation in cholesterol-related diseases [31]. In the following two sections, we will review these two cholesterol metabolism pathways and the influence of LDL and HDL on CD4^+^ T cells.

### 3.1. *De Novo* Cholesterol Synthesis in CD4^+^ T Cells

The key enzyme for de novo cholesterol synthesis is HMG-CoA reductase, which converts HMG-CoA to mevalonic acid (MVA). MVA is converted to squalene via the mevalonate pathway, and squalene can be converted to cholesterol [32].

Statin, a HMG-CoA reductase inhibitor that ameliorates hyperlipidemia, significantly increases the proportion of Treg cells but suppresses Treg cell proliferation [33]. In RA, cholesterol biosynthesis pathway is activated, and Th1 cells treated with statin switch from IFN-*γ*^+^ to IL-10^+^. This indicates that cholesterol biosynthesis is required for the proinflammatory function of Th1 cells [34]. Liver kinase B1 (LKB1) activates the mevalonate pathway. In LKB1-depleted Treg cells, mevalonate enzymes such as HMG-CoA reductase, squalene epoxidase, and acetyl-CoA acetyltransferase are downregulated. Mevalonate pathway activation induces Treg cell proliferation and suppresses IFN-*γ* production [35]. These studies partly explain the relationship between obesity and autoimmune disease. The excessive activation of cholesterol biosynthesis results in the sustained inflammatory activity. Therefore, the clinical approved cardiovascular drug-like statin may be promising in autoimmune disease therapy.

After cholesterol is synthesized, it is usually transported by LDL. The LDL receptor is responsible for cholesterol ingestion by various tissues, especially in liver and blood vessel endothelium. In an LDL receptor-depleted atherosclerosis mouse model, cholesterol accumulates in atherosclerotic plaques. Cholesterol also activates Th1 cell differentiation, indicating its proinflammatory function [36].

LDL can be modified as oxidized LDL (ox-LDL), which causes cholesterol accumulation in the coronary artery, as well as atherosclerosis. *In vitro* studies show that treatment with ox-LDL enables IL-5 to reduce the number of Th1 cells [37]. In an atherosclerosis mouse model, ox-LDL formed immune complexes with circulating antibodies and promoted Th17 cell differentiation and cytokine production [38]. With decreasing plasma cholesterol levels and increasing antibody levels against ox-LDL, the proportion of Treg cells increases, so LDL and cholesterol aggravate inflammation in atherosclerosis [39]. The proportion of Treg cells positively correlates with the abundance of cholesterol, LDL cholesterol, and apolipoprotein (Apo) B in both patients with dyslipidemia and healthy subjects. This is probably due to the protective feedback of Treg cell. Cholesterol and lipoprotein activate such strong inflammation that Treg cell differentiation is also activated but cannot stop the inflammation [40]. ox-LDL also regulates Th17 proliferation through the NF-*κ*B pathway, while it regulates Treg cell apoptosis via the Fas/FasL pathway [41]. ox-LDL is also an important regulator of myeloid cell to induce T cell proliferation and IFN-*γ* production. It depends on MHC-II to activate T cell [42]. These studies suggest that inhibition of LDL can restore T cell differentiation and Th17/Treg balance, which can be used to treat various immune-related diseases.

### 3.2. Ingested Cholesterol in CD4^+^ T Cells

Ingested cholesterol metabolism is the process of reverse cholesterol transport (RCT) to the liver, which is also called HDL metabolism. Smooth muscle cells and macrophages transport cholesterol to HDL [43]. When CD4^+^ T cell activation is inhibited by a lymphocyte-specific protein tyrosine kinase (LCK) inhibitor, the accumulation of cholesterol and macrophages is decreased, and the content of smooth muscle cells is increased in a mouse model of atherosclerosis. Hence, CD4^+^ T cell activation enhances RCT [44].

ApoA-I binds to HDL, which has the potential to reduce the accumulation of lymphocyte cholesterol and lipid oxidation. ApoA-I is also the activator of lecithin cholesterol acyltransferase (LCAT), which catalyzes cholesterol esterification and promotes RCT. HDL cholesterol (HDL-C) exists in the plasma after high-cholesterol diet (HCD). In an HCD mouse model, HCD promoted Th2 cells to produce cytokines, including IL-5, IL-13, and IL-10, indicating that cholesterol may induce Th2 cell differentiation [45]. A similar observation was reported, wherein Th1/Th2 cell numbers were negatively correlated with HDL-C in diabetes [46].

HCD reduces Tfh cell differentiation and accumulation at the germinal center (GC). This reduction is attributed to the fact that HCD enhances PD-L1-mediated suppression of Tfh cell differentiation and immune response [47]. Another study did not observe a significant difference in the proportion of Tfh cells between obese and healthy mice, but it showed that obese mice had more IL-17^+^ Tfh cells and less IFN-*γ*^+^ Tfh cells [48].

High cholesterol levels in Treg cells increase the likelihood of their conversion into Tfh cells, while ApoA-I reduces intracellular cholesterol levels and prevents this tendency [49]. A study recruited 120 patients with diabetes revealed that the proportion of Th1 cells is negatively correlated with HDL-C in diabetes [46]. In mice with normal cholesterol levels, the proportion of Treg cells is positively correlated with plasma levels of HDL-C, suggesting a role of HDL-C in Treg cell homeostasis [33]. On the contrary, ApoA-I does not affect Treg cell activation and migration [50]. Chronic hepatitis C (CHC) is characterized by cholesterol and lipid metabolism alterations. Patients with CHC have increased levels of Th17 cells. After 30 days of a normocaloric low-cholesterol diet (NLCD), patients with CHC showed a significant decrease in the proportion of Th17 cells, as well as increase in the proportion of Treg cells. NLCD inhibits IL-17 and IL-22 production of Th17 cells [51].

Collectively, these results suggest that abnormal cholesterol metabolism can promote the inflammatory effects of Th1 and Th17 cells and inhibit the number and function of Treg cells. Regulating cholesterol metabolism and lipoprotein can not only treat obesity but also inhibit inflammatory autoimmune diseases.

## 4. Prostaglandins in CD4^+^ T Cells

Prostaglandins (PGs) are important immunoregulatory lipid mediators. They have nine subtypes, from PGA to PGI, among which PGC2 and PGH2 are intermediate products. PGD2 binds to exogenous D-prostanoid receptor 1 (DP1), decreasing DP1 activation in CD4^+^ T cells, thereby reducing Th1 cell-associated cytokine secretion in mice [52]. In acute myeloid leukemia mice, PGD2 promotes CD4^+^, CD25^+^, and IL5R*α*^+^ Treg cell proliferation and differentiation, as well as IL-10 production [53].

Cyclooxygenase (COX) is a rate-limiting enzyme involved in the conversion of arachidonic acid to prostaglandin H2, which is the precursor of several molecules, including prostaglandins, prostacyclin, and thromboxanes. PGE2, a product of COX-2, suppresses Th1 cell response. In a study on colitis, a PGE2 inhibitor indirectly induced IFN-*γ* production and T-bet expression in Th1 cells [54]. COX-2 inhibition restores Th1 cell function [55], suppresses IL-17 production, and reduces ROR*γ*t transcriptional levels [56]. COX-1-depleted T cells are less likely to differentiate into Tfh cells, while PGE2 administration restores Tfh cell differentiation [57].

PGE synthase 1 (mPGES1) is a membrane-associated protein that converts PGH2 to PGE2. The deletion of mPGES1 inhibits Th17 cell differentiation in type II collagen-CFA-immunized mouse. Interestingly, exogenous PGE2 also inhibits Th17 cell proliferation [58]. Both in humans and mouse models, prostaglandin E receptor 2- (PTGER2-) *β*-catenin axis serves as a bridge between high-salt diet and autoimmune disease by modulating Treg properties [59]. Moreover, another study showed that high levels of PGE2 suppress IL-1R expression and Th17 cell differentiation via EP4-PKA signaling. PGE2 impairs STAT3 phosphorylation via EP4 activation, which is the receptor for PGE2 [60]. Hydroxy prostaglandin dehydrogenase (HPGD) inactivates PGE2 by converting it to 15-keto PGE2. HPGD expression is upregulated in Treg cells and inhibited by a STAT5 inhibitor or FOXP3 siRNA. The function of Treg cells is impaired after HPGD deletion, which restores PGE2-mediated inflammation and type 2 diabetes [61].

In an allergy-induced asthma model, researchers found that when PGE2 production is suppressed, the Th2 cell transcription factor GATA3 was negatively regulated, indicating that PGE2 is required in Th2 cell activation [62]. PGE2 was also found to induce Tfh cell differentiation in prostate cancer [63]. Several studies reported PGI2 inhibits Th2 cell response in allergic diseases [56, 57]. Thus, different prostaglandin subtypes and prostaglandin receptors can affect the differentiation and function of Th1, Th17, and Tfh cells. Targeting different molecules can be used as clinical treatment for diseases mediated by subsets of CD4^+^ T cells.

Many researchers have focused on COX-PGE2 metabolic pathway and this pathway has been well applied to various therapies, such as aspirin treatment to relieve inflammation. Since much effort has also been made to find out the role of other kinds of prostaglandin, including PGD and PGI, in CD4^+^ T cells, researchers should make more progress in designing more potent and efficacious drugs based on prostaglandin.

## 5. Phospholipid Metabolism in CD4^+^ T Cells

Phospholipids are an important class of lipids on cell membrane that contain two categories, glycerophospholipids and sphingolipids. Sphingomyelin and glycosylphosphatidylinositol (GPI) are components of lipid rafts and play important roles in transmembrane signal transduction. In T cell activation, GPI recruits CD48 to the lipid raft to facilitate the interaction between CD48 and TCR, which activates tyrosine phosphorylation by lymphocyte-specific protein tyrosine kinase (LCK) [64]. CD45 in lipid raft inhibits T cell activation by inhibiting TCR signaling or by integrin-mediated adhesion, while CD45 outside lipid raft promotes T cell activation by constitutively priming LCK through the dephosphorylation of the C-terminal negative regulatory phosphotyrosine site and activating ERK signaling [65, 66]. Therefore, lipid rafts are closely related to the activation of T cells.

Acid sphingomyelinase (ASM) is a lipid hydrolase enzyme that converts sphingophospholipids to ceramides in lysosomes. ASM has the potential to regulate CD4^+^ T cell response [67]. ASM-deficient naive CD4^+^ T cells have a greater probability to differentiate into Th1 and Th17 cells, which were reported to facilitate antitumor immunity in a non-small-cell lung carcinoma mouse model [68]. This may partially be due to ASM deficiency causing a reduction in the production of ceramide, which can be converted to sphingosine-1-phosphate (S1P). S1P significantly reduces the number of CD4^+^ T and Th17 cells [69], but it does not affect Th1 cell differentiation. However, the detail mechanism how S1P regulates T cell differentiation is still unknown [70].

Glycosphingolipids (GSLs) are sphingophospholipids containing glycosyl groups. Sialylated GSLs (gangliosides) on the cell membrane of T cells interact with pathogen antigens. *Shigella*, the enteroinvasive bacterium responsible for acute rectocolitis, binds to CD4^+^ T cell via gangliosides, contributing to T cell activation in the infection mouse model [71]. GM-3, a ganglioside, reduces HIV-1 cell infection by impairing HIV-1 entry [72]. Therefore, regulating GSL metabolism may assist in impairing bacterial or viral infection.

## 6. Conclusions

**Figure 1:**
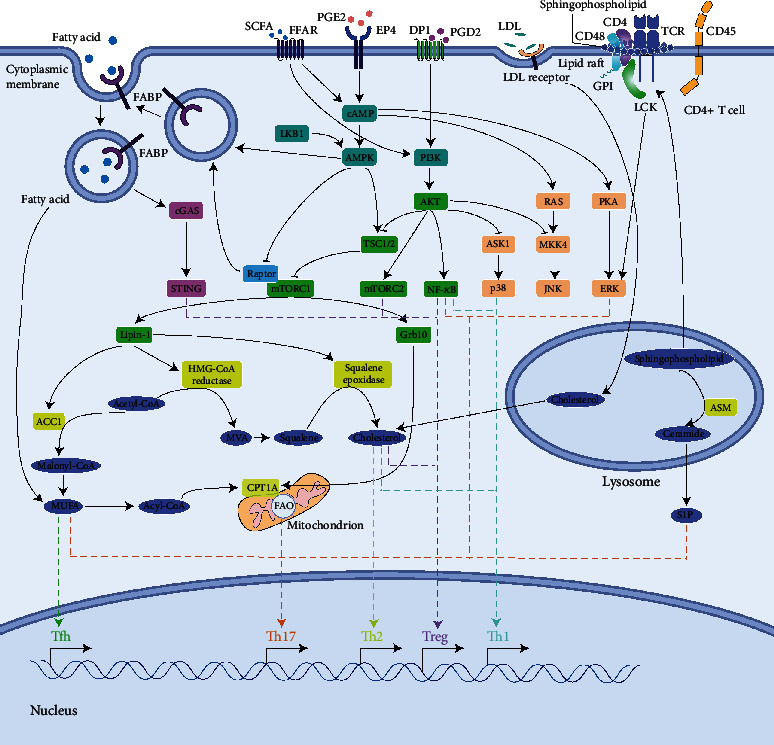
The role of lipid metabolism in CD4^+^ T cell differentiation.Multiple signals affect lipid biosynthesis and degradation. Moreover, lipids, such as fatty acid and cholesterol, also activate multiple signals to regulate T cell differentiation. →: stimulation; ⊣: inhibition.

Lipid metabolism in CD4^+^ T cells is a complicated process and is rapidly gaining importance among researchers. So far, it has been established that lipid metabolism is of vital importance in CD4^+^ T cell activation and differentiation (Figure 1). However, lipid metabolism of T cells is not completely understood. For example, the activation of mevalonate pathway is essential for Treg cell differentiation, while hardly any information is known about de novo cholesterol biosynthesis in Th1 or Th17 cells. With respect to ingested cholesterol, some studies reveal how a surge in the cholesterol level influences T cell function, such as in the cholesterol metabolism caused by HCD. Consequently, the mechanism of ingestion and consumption of cholesterol by T cells, which may be critical in T cell function alteration, has not been studied. Moreover, the lipid raft that promotes TCR and CD28 engagement plays an important role in antigen presentation. Cholesterol and sphingolipid transport is required for lipid antigen presentation [73]. Since several studies in T cells are about cholesterol and sphingophospholipids, which make up lipid rafts, it is possible to clarify the exact function of lipid rafts in T cell activation or T cell-B cell interaction.

Overall, discoveries in the relationship between lipid metabolism and CD4^+^ T cell function will provide novel insights in immune modulation patterns and lead to the identification of new therapeutic targets. Inhibition of effector function and enhancement of Treg cells are essential, with respect to autoimmune diseases and transplant tolerance. Alternatively, targeted metabolism provides a promising new strategy to enhance T cell response in cancer immunotherapy. Further studies are required to find a new therapy based on T cell lipid metabolism regulation.

## Data Availability

The data used to support this review are from previously reported studies and datasets, which have been cited as references.
